# The Ghoul Type of Nurse

**Published:** 1919-02-22

**Authors:** 


### THE MATRONS' AND SISTERS' DEPARTMENT.

### THE GHOUL TYPE OF NURSE.

There are plenty of people, aye, and nurses too, who exclaim with horror at the costliness of the modern nurse training school. What! Fifty thousand pounds for a new nurses' home at the Middlesex Hospital! The thing is preposterous. The protest is due to ignorance of what modern nurse training must include. It arises out of a misconception of the aims to be attained by these elaborately planned establishments. They do not owe their existence to any mistaken desire to make life easy for the lucky probationer and save her from hardships which perhaps other women looking back on old days recognise to have been bracing rather than otherwise. They are planned with a distinct purpose—that of raising the standard of life among those who are to be prepared in them for the career of nursing. The highest type of probationer, it is true, makes good whatever her environment may be during her training. Ordinary young women (and after all, any group of new probationers consists mainly of ordinary young women) need all they can get in the way of stimulus on their road to complete nurses. The majority of them have not thought very seriously about their aims in life previous to entering the hospital. A vague aspiration to do something worth doing has influenced their choice of a calling. This vague aspiration may develop into a fruitful vocation, or it may be stifled in the course of their training. Their surroundings in the nurses' home and the influences brought to bear upon them in the course not only of work, but also of recreation will decide their fate and that of their patients.

Take an ordinary, not conspicuously intelligent young woman of twenty, plunge her into a new world where disease and death in forms strange and startling fill her consciousness, occupy her entire time for almost every hour of every day in the week with duties among the sick of necessity so rapidly performed as to familiarise her with pain, and teach her to regard with indifference the sufferings she witnesses; let the only events of the day be the events of the ward; isolate her from books, music, and beautiful things; give her no counsels about the control of her thoughts, nor any counteracting aids to check the growth of morbidness in her outlook; make outside friendships impossible; let her endless round of duties, for the most part mechanical, lift only when she tramps the streets or (rare luxury) has tea in a shop; feed her mind remorselessly with scraps of physiology, bearing always, always, on diseased conditions; drive her to find relief for over-taxed nerves solely in scandal or the gruesome gossip of the hospital as it filters through from one to another, with whispered jokes on forbidden matters. Such is the recipe for manufacturing the Ghoul nurse. The public has complained long enough of this terror in the sick-room. Her existence is notorious. The modern nurse-training school is designed to shut off the source from which she springs.

If the group of new probationers strikes the observer as very ordinary, there is at least this to be said about it. It consists of thoroughly normal women. The process of filtration has been reduced to fine art by the modern matron, accustomed to read character as an open book. If she fails in her diagnosis she has either the resource of the preliminary training-school, a searching test, or that of the trial two or three months under different ward sisters. This suffices to weed out the neurotic, the densely stupid, the perverse, who may have slipped through the mesh. There remains a squad of women all intensely anxious to prove their fitness for their vocation, sound in health, capable of responding to instruction. This accidental group of applicants is as hopeful a set as is likely to be discovered by any process of selection which could be devised. They are malleable, unprejudiced, eager. Their future as nurses depends on how they are handled.

Who is altogether satisfied with the average type of nurse now being turned out? Immeasureably as the standard has been raised during the last twenty years even, the improvement renders the demand for further progress more insistent. It is just because the public has learned to rejoice in the ministrations of sympathetic, discreet, self-controlled nurses that they regard with impatience and loathing the ghoul type who fasten upon and tattle about every sad detail of the sick-room, terrify their patients with forebodings, and disgust them by the revelation of a mind wholly mastered by the influences they exist to combat.

If we would teach the probationer to master these influences from the first moment she encounters them, if we would counteract the associations with disease to which she is perforce exposed, the modern nurse training school is a necessity. But it is not cheap.

